# A Preliminary Exploratory Study of Autologous Fat Transplantation in Breast Augmentation With Different Fat Transplantation Planes

**DOI:** 10.3389/fsurg.2022.895674

**Published:** 2022-06-10

**Authors:** Bin Li, Yuping Quan, Yufei He, Yunfan He, Feng Lu, Yunjun Liao, Junrong Cai

**Affiliations:** Department of Plastic and Cosmetic Surgery, Nanfang Hospital, Southern Medical University, Guangzhou, China

**Keywords:** autologous fat transfer, breast augmentation, pectoralis major, oil cysts, inflammation, macrophage

## Abstract

**Background:**

Autologous fat transfer is common in breast augmentationor reconstruction. However, AFG recipient site in the breast for fat grafting has not been carefully investigated.

**Methods:**

Forty female patients requiring breast augmentation with fat grafting were randomly assigned into two groups. The retromammary group received 2/3 fat into the retromammary space and the other 1/3 into the subcutaneous and retropectoral planes. The retropectoral group received 2/3 fat into the retropectoral plane and the other 1/3 into the subcutaneous and retromammary planes. The fat grafting result at 6 months was assessed by 3D laser surface scanning and then ultrasound. Any complications were recorded during follow-up. Samples from a patient who underwent fat grafting for 6 months was obtained and histological examination was conducted.

**Results:**

No significant difference in the retention rate after 6 months was observed between the two groups (retromammary group: 35.9% ± 6.6; retropectoral group: 39.3% ± 5.1, *p* = 0.1076). The retromammary grouphad a higher incidence of oil cyst formation than the retropectoral group. Histological examination showed that there were more oil cysts and mac2 positive macrophage infiltration in the fat cells in retromammary group, while retropectoral group had more small-size adipocytes.

**Conclusion:**

Although fat grafting into the retropectoral plane did not provide a superior fat graft retention rate, it did lower the incidence of complications. The retropectoral space show great potential to become a favorable recipient site.

## Introduction

Autologous fat is considered to be an ideal soft tissue filling material for both cosmetic and reconstructive purposes, and has been used extensively in plastic surgery (1–3). Fat grafting is increasingly used for cosmetic breast augmentation (4). Currently, fat graft retention is more predictable and ideal using standardized techniques (5–7), but complications that occur after breast augmentation with fat grafting have made it controversial for wider use and further study is required (8).

Breast fat grafting can lead to complications, including infection, seroma, palpable cysts, and calcification, which concerns both surgeons and patients (9–12). Various factors can affect the fat grafting outcome, such as fat processing method, injection volume, and patient health status (13–15). The injection site is likely to be among these factors; however, it has not yet been studied.

Recipient sites in the breast that can be selected as the injection planes for fat grafting: beneath the skin, mammary gland, or the pectoralis major. To date, no consensus has been reached as to the optimum injection plane. The retrothoracic plane is usually used to place breast implants in cosmetic and reconstructive surgery. This site is scarce in adipose tissue, which is vascularized by contact with the pectoralis major andminor muscles and intercostal muscles (16). To date, there are no reports of fat graft outcome when injected beneath the pectoralis major.

In this study, we conducted an exploratory study to evaluate efficacy and safty of autologous fat transplantation in breast augmentation with different fat transplantation planes. We used 3D laser scanning to assess fat graft outcome. Additionally, we obtained samples of retropectoraland retromammary surviving fat from the breasts of patients who underwent autologous fat transplantation for breast augmentation.

## Materials and Methods

### Study Design

A preliminary exploratory study on different fat transplantation planes in breast augmentation was made. Forty patients were included in this study and underwent autologous fat grafting for breast augmentation. Since it is an exploratory study, the sample size is not calculated based on statistical principles. These patients were randomly assigned to either the retromammary or the retropectoralgroup.

### Patients

This study was conducted between September 2019 and October 2020. Inclusion criteria were: healthy adult women, body mass index (BMI) <25 kg/m^2^, BI-RADS score ≤2. Exclusion criteria were: breast disease (e.g. fibrocystic breast disease and mastitis), diseases of the immune system, and other diseases making surgery unsuitable. This study was approved by the institutional review board of Nanfang Hospital, Southern Medical University, and was conducted in accordance with the guidelines of the Declaration of Helsinki. Each participant received detailed oral and written information about this study, including the risks, beneﬁts, and alternative therapies, and each signed an informed consent form.

### Surgical Methods

Fat tissue was obtained from the patient’s thigh and/or abdomen. Blunt head liposuction needles (needle tube diameter 3.0 mm, side hole diameter 2.0 mm) and 10 mL screw mouth syringe were used for low negative pressure suction. Lipoaspirates were put into a centrifuge and spun at 1,200 *g* for 3 min. After centrifugation, the oil layer (upper level) was decanted and the aqueous layer (lower level) was drained out of the syringe. The middle layer, predominantly composed of fat grafts, was used for the present study (17).

In the retromammary group, 2/3 of fat was injected in the retromammary plane while the residual half in subcutaneous plane and retropectoral plane. In the retropectoral group, 2/3 of fat was injected in the retropectoral plane while the residual half in subcutaneous plane and retromammary plane. Fat was injected in the retropectoral plane when cannula was inserted close to the rib surface. To assess the retromammary plane, cannula was inserted upward into the area where glands locate. It was easy to touch the cannula when inserted in subcutaneous plane.

To get access to the retropectoral plane, the intersection of the inframammary fold and the anterior axillary line was chosen as the insertion point, and the lipoinjection cannula was inserted above the ribs and intercostal muscles. To inject lipoaspirates into the retromammary plane, the lipoinjection cannula was inserted at the midline of inframammary fold and the breast was lifted up to enlarge the retromammary space.

### Evaluation of Results

At baseline (pre-operation) and 6 months post-operation, photographs were taken by the same photographer in a studio with consistent camera settings, lenses, seating position and lighting. 3D laser surface scanning (MVS-600; CASZM, Shenzhen, China) was also performed with a portable device to objectively calculate the volume of the breasts. The scanning process lasted less than 60 sec and involved multiple viewpoints. Data from these scans were merged for volumetric analysis using software “ZKZM 3D Analyze” (CASZM, Shenzhen, China) on a computer, and each breast per patient was blindly analyzed pre-treatment, and 6 months post-treatment.

### Postoperative Complications

Possible complications, including post-operative hematoma, infection, calcification and oil cysts, were assessed by ultrasound scanning (ACUSON Sequoia, Siemens). Oil cysts >10 mm in diameter, as determined by ultrasound, were compared between the two groups of patients. Some patients who required magnetic resonance imaging (MRI) examination were also examined before and 6 months after the breast operation. A 1.5 Tesla scanner (Avanto; Siemens, Germany) was used with 3-mm thick slices.

### Sample Harvest and Histologic Evaluation

One patient, who underwent breast augmentation with implant 6 months after autologous fat grafting, provided approval to obtain the transferred fat tissue during the implant surgery. Adipose tissue was obtained beneath the skin and pectoralis major. The tissue was embedded in paraffin, sectioned, and stained with hematoxylin and eosin (HE) staining according to standard protocols. Immunofluorescence staining was performed with rabbit anti-human Perilipin (ThermoFisher, USA) and mouse anti-human Mac-2 (Abcam, USA) primary antibodies.

### Statistical Analysis

For analysis, data were imported into IBM SPSS Version 23.0 software (IBM Corp., Armonk, N.Y.). Statistical testing was performed with values of *p* < 0.05 considered as statistically significant by means of two-sided testing. Continuous variables were compared using a t-test. Non-normal data were tested for rank equality between strata by Mann-Whitney U tests.

## Results

### Patient Characteristics

Table 1 shows the demographic and clinical characteristics of the patients. All 20 patients in the retromammary group were non-smoking women with a mean age of 28.0 ± 4.7 years. The mean BMI of these ten patients was 21.6 ± 2.4 kg/m^2^. The mean injected volume of fat tissue was 307.5 ± 30.4 mL per breast. All 20 patients in the retropectoral group were non-smoking women with a mean age of 29.7 ± 4.9 years. The mean BMI of these 10 patients was 20.4 ± 2.3 kg/m^2^. The mean injected volume of fat tissue was 311.5 ± 20.2 mL per breast. The two groups were well-matched, with no significant differences in age, BMI, or injected volume (*P* > 0.05 each).

**Table 1:** Summary of patient data.

	Retromammary	Retropectoral	*p*
Total cases	20	20	N/A
Age (year)	28.0 ± 4.7	29.7 ± 4.9	0.441
BMI (kg/m^2^)	21.6 ± 2.4	20.4 ± 2.3	0.527
Injected volume/per breast (mL)	307.5 ± 30.4	311.5 ± 20.2	0.478
Donor Site			
Abdomen	8	8	N/A
Thighs	10	8	N/A
Abdomen and thighs	2	4	N/A

### Evaluation of Fat Graft Retention

**Figure 1:**
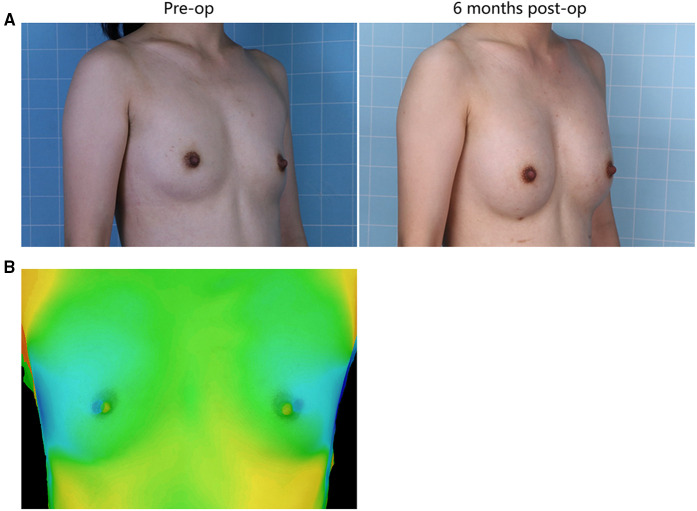
(**A**) A 28-year-old female patient from the retromammary group presented with involutional atrophy of the breasts. She received 330 mL lipoaspirates per breast, with 220 mL into the retromammary space, 55 mL into the subcutaneous plane and 55 mL into the retropectoral space. Six months after surgery, each breast was augmented. (**B**) The 3D laser scanner demonstrated the volume change before and after surgery.

**Figure 2:**
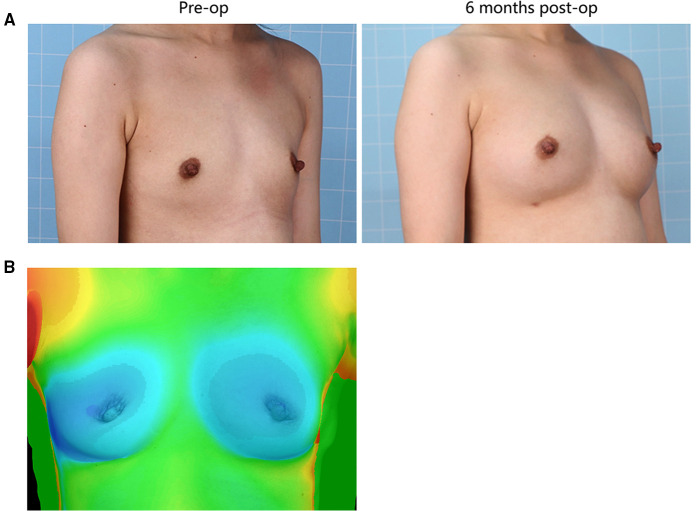
(**A**) 25-year-old female patient from the retropectoral group presented with involutional atrophy of the breasts. She received 300 mL lipoaspirates per breast, with 200 mL into the retropectoral space, 50 mL into the subcutaneous plane and 50 mL into the retromammary space. Six months after surgery, the breasts gained fullness and a well-defined shape. (**B**) The 3D laser scanner demonstrated the volume change before and after surgery.

**Figure 3:**
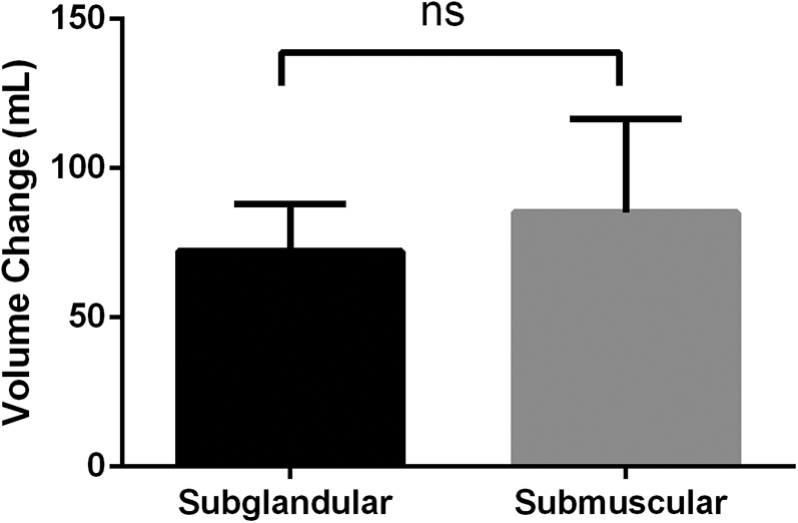
The mean retention rate of the fat graft in the retromammary group after 6 months was 35.87% ± 6.60, and 41.28% ± 7.64 in the retropectoral group. No significant difference was observed between these two groups (*p* = 0.1076).

Illustrative images of both groups demonstrated the improved breast fullness and well-defined breast contour after fat grafting for 6 months (Figures 1, 2). The mean retention rate of the fat graft in the retromammary group after 6 months was 35.9% ± 6.6, and 39.3% ± 5.1 in the retropectoral group (Figures 1, 3). No significant difference was observed between these two groups (*P* = 0.1076).

### Complications

During follow-up, no patient in either group experienced adverse events, such as calcificationor hematoma (Table 2). One patient in the retromammary group had infection, while no patient in retropectoralgroup had infection (*P* > 0.05). Ultrasound examination showed that oil cysts were present in all patients of both groups, although their number and size differed significantly between the two groups. In the retropectoral group, the majority of the cysts were <1 cm in diameter and only one patient had an oil cyst >1 cm in diameter. By contrast, oil cysts in the retromammary group were generally larger in size. Six patients in the retromammary group developed cysts >1 cm in diameter (*P < 0.05).

**Table 2:** Complication rate.

	Retromammary	Retropectoral	*p*
No. of patients	20	20	N/A
Oil cyst (>1 cm)	12	2	0.033
Infection	2	0	0.305
Calcification	0	0	N/A
Hematoma	0	0	N/A

### Histologic Observation

**Figure 4:**
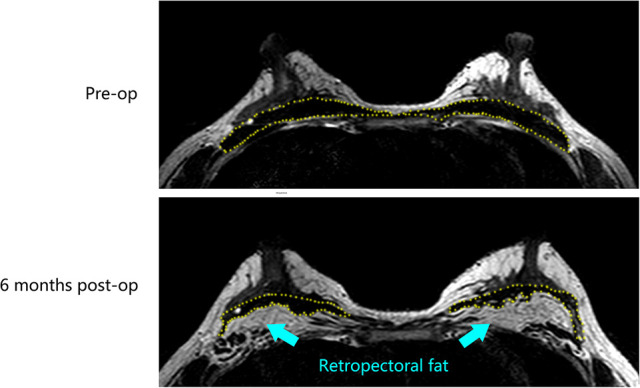
Magnetic resonance images show that a thick fat pad has survived under the pectoral major 6 months after fat grafting. The yellow-dotted line indicates the border of the pectoral major muscle.

**Figure 5:**
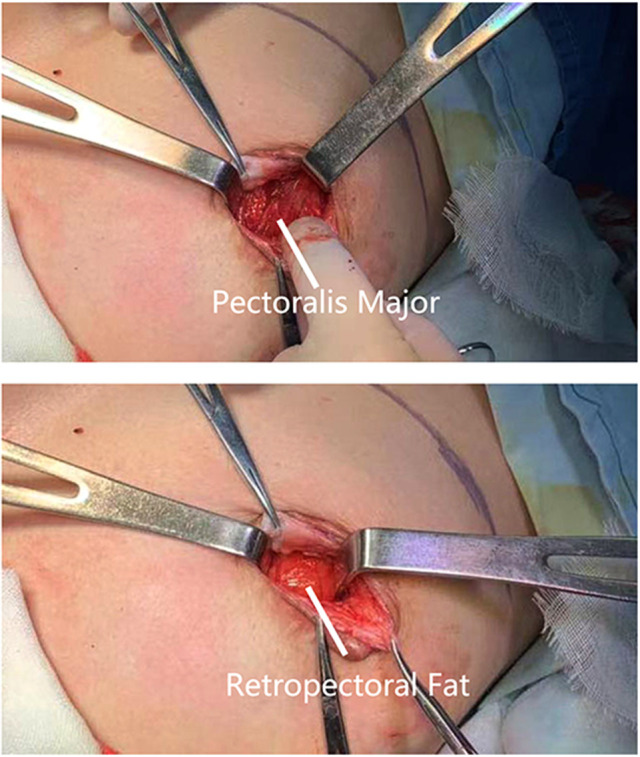
Collection of fat specimen during an implant-based breast augmentation 6 months after fat grafting. During the surgery, the transplanted fat tissue was clearly observed beneath the pectoralis major muscle.

**Figure 6:**
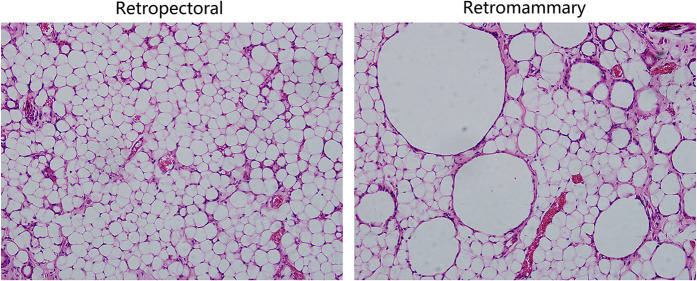
Hematoxylin and eosin (HE) staining results of the retromammary and retropectoral fat. (Left) HE staining results of retropectoral fat tissue. The morphology of the adipocytes is uniform, and few oil cysts can be observed. (Right) HE staining results of the retromammary fat tissue. Oil cysts with different size can be observed in the surviving fat tissue.

**Figure 7:**
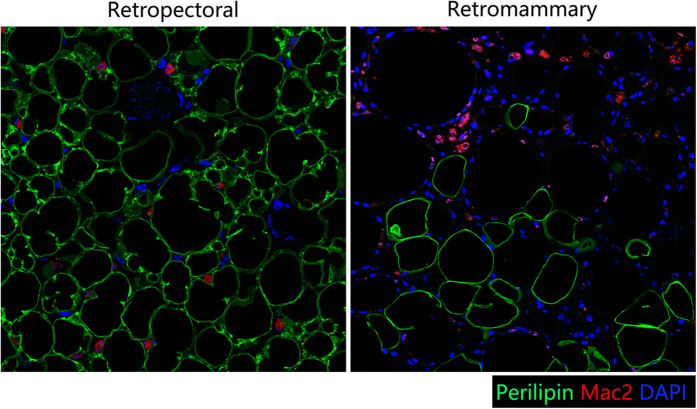
Immunofluorescence staining of perilipin and MAC-2. (Left) In the retropectoral transferred fat sample, several multilocular adipocytes and few MAC-2 positive (+) macrophages were found. (Right) In the retromammary transferred fat sample, less adipocytes and more MAC-2+ macrophages were found.

A28-year-old female patient who underwent breast augmentation with implant 6 months after autologous fat grafting provided approval for us to obtain the transferred fat tissue during their implant surgery. Before implant surgery, her MRI results indicated that a thick fat pad had survived under the pectoral major (Figure 4). During the surgery, the transplanted fat tissue was clearly observed after opening the pectoralis major during the implant operation (Figure 5). Retropectoraland retromammary adipose tissue were obtained for histological examination. The retropectoralsample had smaller sized adipocytes and less inflammatory cell infiltration than the retromammary one. In the retromammary transferred fat tissue, numerous oil cysts were found, while few were found in the retropectoralfat graft (Figure 6). The expression levels of perilipin and Mac-2 were detectedby immunofluorescence. In the retropectoralsample, several multilocular adipocytes and few MAC-2 positive (+) macrophages were found. In the retromammary sample, less adipocytes and more MAC-2+ macrophages were found (Figure 7).

## Discussion

Despite the increasingly widespread use offat transfer in plastic surgery, there is no superior anatomical plane in the breast for fat grafting that is accepted in the literature. This study aims to evaluate the clinical outcome of fat grafting into different injection planes for breast augmentation. The three spaces (subcutaneous, retromammary, and retropectoral) in breasts were selected as the injection planes in our study. Subcutaneous space is a suitable fat grafting plane because it is where adipose tissue naturally grows; however, this space has too little room to contain enough lipoaspirates for augmentation. The retromammary and retropectoral spaces have moreroom and are commonlywhere implants are placed for breast augmentation (18–20). In implant-based breast augmentation, the retromammary space was the first plane described and still frequently used (21). The retropectoral pocket, introduced in 1968, was refined by the division of the costochondral origins of pectoralis major and has become increasingly popular (22, 23). Evidence from implant surgery indicates that fat grafts should ideally be placed in either of these two spaces to allow enough room for augmentation.

Our study first divided the patients into two groups. In both groups, lipoaspirates were injected into the three layers (the subcutaneous, retromammary, and retropectoral spaces), but were distributed in different proportions. The retromammary group received 2/3 lipoaspirate into the retromammary space, while only 1/6 was injected into the retropectoral plane. The retropectoral group received 2/3 lipoaspirate into the retropectoral space, while only 1/6 was injected into the retromammary plane. The 3D laser scanner was used to evaluate fat graft retention rate. The results suggested that the graft retention rate in the retropectoral group was slightly higher than in the retromammary group, but no statistical difference was observed (*p* > 0.05). MRI results also clearly showed that the fat graft could survive well in the retropectoral plane. It is believed that blood circulation at this site is less pronounced than in the intraparenchymal space, and that movement of muscle would compromise the retention rate of fat grafts. Therefore, the retropectoral plane was not recommended for fat grafting by most surgeons. However, our results suggested that retropectoral fat graft retention was not compromised, and was even slightly higher than in the retromammary group, though no statistic difference was found.

Few studies have provided data on fat graft retention in the retropectoral plane. One study showed that the mean fat graft retention rate in the retropectoral plane was 23.4% in the late post-operative period (24). The mean retention rate in our study was 39.28% and thus higher than their result. Indeed, these two results are not comparable, because of uncontrolled, potentially interfering factors, including fat processing methods, injection volume and volume distribution in different injection planes.

Although the two patient groups shared similar retention rates, the retropectoral group had significantly fewer complications than the retromammary group. Large oil cysts were not observed in any patient in the retropectoral grafting group after surgery. By contrast, two patients in the retromammary group developed palpable nodules, and two patients developed oil cysts up to 5 cm in size. The occurrence of large oil cysts was significantly lower in the retropectoral than in the retromammary group, indicating that fat grafting into the retropectoralplane reduced the development of oil cysts, while fat grafting into the retromammary plane had a higher risk for oil cyst formation. Ultrasound scanning (ACUSON Sequoia, Siemens) was used to assesse the possible complications, including post-operative hematoma, infection, calcification and oil cysts in all patients. No distinct changes were observed in the BIRADS classification in 6 months post-operation. Masaaki et al. analyzed the ultrasound results of 256 patients after fat grafting and found that 77% of the breasts had lumps located beneath the mammary glands, 17% beneath the skin, and 6% beneath the pectoralis major muscle (25, 26).

Currently, most researchers use animal models to investigate the regenerative mode of fat grafts due to difficultly in accessing the human samples. In this study, one patient who underwent implant breast augmentation 6 months after autologous fat grafting allowed us to access a human sample of fat grafts. Because there is no fat on the surface of (retromammary) or beneath (retropectoral) the pectoralismajor under normal conditions (27, 28), the retromammary and retropectoral spaces were selected to be sampled in this study. Therefore, we harvested the surviving faton the surface of and beneath the pectoralis major. The histological results showed that the retropectoral fat graft had a healthy adipose tissue structure. Few oil cysts or MAC-2+ macrophages were observed in the retropectoral fat graft.

In theory, muscular tissue, as it is more vascularized, may be a protective factor for AFG and increase fat intake when compared to AFG in the subcutaneous/subglandular space (29). In the immunofluorescence results, numerous multilocular adipocytes were observed in the retropectoral fat graft. These may be newly-born adipocytes or beige adipocytes. Since the sample was harvested at 6 months post-fat-grafting and regeneration should have finished, we assumethat these adipocytes were of the beige type. Recently, screening of muscle cells over-expressing PGC-1α has led to the identification of PGC-1α-dependent myokines involved in promoting beige fat thermogenesis, includingirisin, β-aminoisobutyric acid (BAIBA), myostatin, and FGF-21 (30). Therefore, the pectoralis major muscle may induce browning of the retropectoral fat graft. Previously, we showed that tamoxifen-induced browning of adipose tissue could improve fat graft quality and retention rate (31). Zhu et al. also reported that supplementation with extracellular vesicles derived from adipose-derived stem cells promote beige adipose regenerationand increases fat graft survival (32). The browning of transferred fat induced by the pectoralis major muscle would probably contribute to the fat graft retention improvement.

The limitation of this study is that it is an exploratory study with a small sample size and the results obtained are not robust. In addition, this study lacks long-term follow-up data, which makes this study unable to provide information on other complications, such as embolism, calcification and long-term retention rate. Moreover, the volumetric method applied in our study is the use of a laser scanning instrument and 3D analysis. Though it is an economical and reproducible method, measurements of volume changes with the 3D analysis might not be as accurate as CT and MRI. Later, we plan to conduct a larger sample size and more scientific and rigorous confirmatory study to demonstrate the conclusions of this study.

## Conclusion

This exploratory study indicates that compared with retromammary fat grafting, retropectoral fat grafting had a similar fat graft retention rate and significantly lower complication rate. The morphology of the adipocytes in the retropectoral fat is uniform, and few oil cysts were observed. The retromammary fathad more oil cysts and macrophage infiltration. The retropectoral space show great potential to become a favorable recipient site, but further study is required to verify.

## Data Availability

The original contributions presented in the study are included in the article/Supplementary Material, further inquiries can be directed to the corresponding author/s.
